# Excellence in Family Paediatricians: the FIMP-MCRN (Medicines for Children Research Network) becomes a member of ENPR-EMA (European Network of Paediatric Research at the European Medicines Agency)

**DOI:** 10.1186/1824-7288-37-7

**Published:** 2011-01-19

**Authors:** Ettore Napoleone

**Affiliations:** 1 Piazza della Vittoria 14/A Campobasso 86100 ITALY

## Abstract

One of the objectives of the Paediatric Regulation (EC) No 1901/2006, is to foster high quality ethical research on medicinal products to be used in children. To achieve this objective, the EMA is responsible for developing a European paediatric network of existing national and European networks and centres with specific expertise in research and clinical trials relating to paediatric medicines. The purpose of this article is to disseminate knowledge of the structure and goals of ENPR-EMA and to highlight the cultural and organizational difficulties for its implementation.

Following the publication of research quality requirements, a set of recognition criteria, which have to be fulfilled to become a member of ENPR-EMA were agreed. So far, 32 networks and centres (of 62 identified networks) submitted self-assessment reports indicating whether or not they fulfill the agreed minimum criteria. Sixteen networks (26% of 62 identified networks) fulfilled all minimum criteria and became therefore members of ENPR-EMA. The Family Paediatricians Medicines for Children Research Network (FIMP-MCRN), established with the aim of developing competence, infrastructure, networking and education for paediatric clinical trials, became member of the ENPR-EMA responding satisfactorily to all the points of the self-assessment report.

## Introduction

One of the objectives of the Paediatric Regulation (EC) No 1901/2006, is to foster high quality ethical research on medicinal products to be used in children [1,2].

This Regulation aims to facilitate the development and accessibility of medicinal products for use in the paediatric population, to ensure that medicinal products used to treat the paediatric population are subject to ethical research of high quality and are appropriately authorized for use in the paediatric population, and to improve the information available on the use of medicinal products in the various paediatric populations. These objectives should be achieved without subjecting the paediatric population to unnecessary clinical trials and without delaying the authorisation of medicinal products for other age populations [1].

A scientific committee, the Paediatric Committee, within the European Medicines Agency with expertise and competence in the development and assessment of all aspects of medicinal products to treat paediatric populations [1].

The Paediatric Committee is primarily responsible for the scientific assessment and agreement of paediatric investigation plans and for the system of waivers and deferrals thereof; it should also be central to various support measures contained in this Regulation. In its work, the Paediatric Committee consider the potential significant therapeutic benefits for the paediatric patients involved in the studies or the paediatric population at large including the need to avoid unnecessary studies.

Clinical trials in the paediatric population may require specific expertise, specific methodology and, in some cases, specific facilities and should be carried out by appropriately trained investigators. A network, which links existing national and Community initiatives and study centres in order to build up the necessary competences at Community level, and which takes account of Community and third country data, would help facilitate cooperation and avoid unnecessary duplication of studies [1].

This network should contribute to the work of strengthening the foundations of the European Research Area in the context of Community Framework Programmes for Research, Technological Development and Demonstration Activities, benefit the paediatric population and provide a source of information and expertise for industry[1].

The Article 44 of the Paediatric Regulation (EC) No 1901/2006 included [1]:

1. The Agency shall, with the scientific support of the Paediatric Committee, develop a European network of existing national and European networks, investigators and centres with specific expertise in the performance of studies in the paediatric population.

2. The objectives of the European network shall be to coordinate studies relating to paediatric medicinal products, to build up the necessary scientific and administrative competences at European level, and to avoid unnecessary duplication of studies and testing in the paediatric population.

## ENPR-EMA (European Network of Paediatric Research at the European Medicines Agency)

The European Paediatric Regulation calls for the fostering of high-quality, ethical research on medicinal products to be used in children. To achieve this objective, the EMEA is responsible for developing a European paediatric network of existing national and European networks and centres with specific expertise in research and clinical trials relating to paediatric medicines. The purpose of this article is to disseminate knowledge of the structure and the goals of ENPR-EMA and to highlight the cultural and organizational difficulties for its implementation.

## Detailed goals of ENPR-EMA [3]

The short term and long term goals of the network encompass:

### Collaboration

- identify, co-ordinate and link together existing networks

- ensure efficient, timely communication and exchange of information between networks inside and outside the EU, including with WHO

- be a source of information and expertise for health professionals

- provide a forum for scientific discussion related to paediatric clinical trials with all stakeholders, where necessary

### Building competences

- define scientific and operational quality standards, and recognition criteria by networks themselves for the purpose of the operation of the network in particular not yet covered by existing quality standards (e.g. GCP)

- develop and agree training and education curricula and provide training to network partners

- stimulate the development of new networks, centres and investigators

- organise and hold scientific meetings to discuss specific topics as identified by the Coordinating Group

- stimulate research on trial methodology and educate

### Avoiding unnecessary studies

- avoid duplication of clinical trials in children by sharing information with European as well as international partners, in particular through the use of EudraCT develop multidisciplinary research partnership

### Stimulating high quality research

- raise awareness on the need for clinical trials for children and increasing understanding of the purpose of research

- contribute to GCP compliance

- advocate for ethical clinical research, especially when trials include patients outside the EU.

### Strengthening the foundation of the European Research Area

- Support development and research into off-patent medicines for children, including contribution to priority list of off-patent medicines

- stimulate research in areas of relevance such as trial methodology or non-invasive assays

### Facilitation of implementation and recruitment of clinical trials

- enable rapid attainment of sample sizes large enough to allow valid conclusions through effective network collaboration and facilitation of performance of trials across Member States; this is especially relevant and crucial for paediatric trials.

## Steps for ENPR-EMA organisation

On 16 February 2009 the EMEA convened a one-day workshop to discuss and initiate the development of this European paediatric network. Following a call for expression of interest, 62 networks and/or clinical trial centres have been identified [4].

In the first session, (38 network) participants were reminded of the European Paediatric Regulation and the objectives of this EMEA European network, and the role of the Paediatric Committee (PDCO). The link with the paediatric investigation plans, the extent of clinical trials proposed and the wide range of therapeutic areas addressed so far by the PDCO were highlighted, as it is expected that the network will be involved in performing the trials requested by the Paediatric Investigation Plans (PIP).

In the second sessions the proposed organisation and structure of the future European paediatric network were presented as laid out in the Implementation strategy, adopted by the EMEA Management Board following a large consultation process.

Two break-out sessions were held to brainstorm and discuss the possible structure and operational model for the European network as well as communication strategies (Group 1), and quality standards and recognition criteria (Group 2).

Group 1 discussed the composition of the future "Coordinating Group" as proposed by the implementation strategy. The participants of this break-out session concluded that the coordinating group should aim at being as diverse as possible, representing various types of networks: on specific therapeutic areas, on specific needs/age subsets or specific activities and organizational, transversal networks to cover all areas of paediatric research. It was discussed that clinical trial centres are usually more interested in phase I-III studies whereas a network able to mobilise community paediatricians would be more appropriate for the conduct of long-term follow-up, phase IV studies.

With regards to communication, the need and importance for external communication with all stakeholders (industry and patients' organisations) was stressed. Relating to internal communication some participants reported from their experience that in addition to time-saving communication ways, such as e-mails, telephone and/or videoconferences, at least one or two meetings per year, inviting all members, proved to be helpful and were highly appreciated to overcome potential misunderstandings due to language barriers and different cultures across Europe.

Group 2 was tasked to define "recognition criteria". Proposals for recognition criteria included:

• Capacity to involve patients from both the design point of view and the recruitment

• Expertise in the therapeutic area

• Capacity to manage trials and to perform according to GCP

• Capacity to build up competence and involve further centres

• Capacity to innovate in trials (e.g. methodology, use of microassays)

• Established quality assurance of the network

• Potential conflicts of interest

It was considered necessary to first precise the goals of the European network and the topics on which networks want to work together [4].

On 16 March 2010 the EMA convened a one-day follow-up workshop on the European paediatric research network. Following the outcome of the first workshop with participants of 38 networks and/or clinical trial centres in February 2009, two working groups with members of identified networks were established and were tasked to elaborate:

1. The structure for the operation of the European Paediatric network.

2. Recognition criteria for existing networks which will have to be fulfilled to become a member of ENPR-EMA (the European Paediatric Research Network at the EMA) [3,5,6].

The aim of the second workshop was to discuss with the networks the proposals elaborated by the two working groups, and to come to an agreement on the recognition criteria and the structure for the operation of ENPR-EMA. Twenty-two networks were represented by 27 participants.

The first session was dedicated to discussing the structure for the operation of ENPR-EMA. The implementation strategy for ENPR-EMA adopted by the EMA Management Board proposed to create a 'Coordinating Group' which would contribute to the short and long-term strategy of the network, discuss and solve operational and scientific issues for the network, report to the Paediatric Committee and act as a forum for communication.

## "Coordinating Group"

The chair of Working Group 1 presented their proposal for the composition of the Coordinating Group:

• The Coordinating Group should a) be as diverse as possible, b) inclusive to represent various types of networks: networks focusing on specific therapeutic areas, specific needs/age subsets (e.g. neonatal/adolescent networks) or specific activities (e.g. pharmacovigilance), as well as organizational, transversal networks (e.g. national networks linking together either several clinical trial centres or community paediatricians), accommodating for regional differences throughout Europe with regards to how the medical care of children is organised.

• According to the implementation strategy the total number of members shall not exceed 20, including one member representing the EC and 2 members of the Paediatric Committee. Thus the Coordination Group will consist of 18 members of existing and recognised networks. The working group could not agree that initially all networks meeting the recognition criteria would automatically become members of the Coordinating Group, whereas other networks would then have to group themselves to be represented, once the maximum number had been reached.

## The composition of the Coordinating Group

• 4 networks representing national networks

• 10 members representing diverse therapeutic areas:

- Oncology/Haematologic Malignancies

- Diabetes/Endocrinology/metabolic disorders/Gynaecology

- Gastroenterology/Hepatology

- Allergology/Immunology/Rheumatology

- Stem Cell and Organ Transplantation/Haematology(non malignant)/Haemostaseology

- Respiratory diseases/Cystic Fibrosis

- Cardiovascular diseases/Nephrology

- Psychiatry/Neurology

- Infectious diseases/Vaccinology

- Intensive Care/Pain/Anaesthesiology/Surgery

• 4 members representing special activities/age groups:

- 1 member from European neonatal network

- 1 member representing European paediatric pharmacists

- 1 member representing special activities, eg pharmacovigilance, long-term follow up, Phase 4 studies eg via network of community paediatricians

- 1 member representing expertise in clinical trial methodology. This member could also represent networks not actually performing clinical trials (such as TEDDY, PRIOMEDCHILD).

## The tasks of the Coordinating Group

The main tasks identified were:

- to facilitate access for industry to paediatric clinical study sites (e.g. coordinate industry requests/enquiries/feasibility) to the Networks/Centres of Excellence/experts/societies.

- to act as a platform to communicate and negotiate with industry

- to work on agreement/contracts with pharmaceutical industry regarding paediatric trials

- to identify networks which are not yet on the Agency list

- to develop common educational tools for patients/parents to increase willingness to participate in paediatric trials

- to help ensure feasibility of studies and monitor trial recruitment so that feasibility can be maintained [3,5,6].

## Recognition criteria and self-assessment report

The chair of Working Group 2 informed the participants how the criteria were elaborated, using both the Delphi technique for the first and second round, and the Nominal group technique in the face-to-face meeting held in December 2009 at the EMA [7].

In the first round of the Delphi survey, all 62 networks identified were asked for suggestions of recognition criteria. Thirty replies (30/62, 48%) were received including one from EMA. Similar proposals were grouped within 8 categories in order to obtain a list of the most cited criteria. Only criteria that can be quantified, either qualitatively (e.g. yes or no or other scales) or quantitatively (e.g. numbers) were taken into account.

In the second round, sent again to the 62 networks, responders were asked to rank the 8 categories from 8 for the most important, to 1 for the least important. Within each of the 8 categories, responders were asked to select one or several essential items that are important to quantify the related category.

The response rate to the second round was 45/62 (73%): 41 completed the survey, 4 refused and 17 did not reply. Based on these responses, the members of both working groups were invited to a consensus conference at EMA with the goal to establish the final list of criteria. During this meeting the final list was condensed to the following 6 categories, each with several sub-categories:

1. Research experience and ability (13 subcategories)

2. Network organisation and processes (10 subcategories)

3. Scientific competencies and capacity to provide expert advice (7 subcategories)

4. Quality management (7 subcategories)

5. Training and educational capacity to build competences (5 subcategories)

6. Public involvement (3 subcategories)

The last part of the workshop was dedicated to defining minimum recognition criteria to become a member of ENPR-EMA. There was general consensus on a definition of a minimum set of criteria based on the following:

- Recognition criteria are related to quality of research

- A minimum level of quality must be provided

- The intention is to be open to new/emerging networks and organisations.

The minimum requirements agreed were:

At least 1 ongoing or completed trial (Criterion 1)

An identified contact person (Criterion 2)

An external or internal Advisory Board (Criterion 2)

Internal patient database or disease registry (Criterion 2)

Individual data protection and data security (Criterion 2)

Access to experts/expert groups (Criterion 3)

Capacity to answer scientific questions (Criterion 3)

Compliance with GCP (Criterion 4)

Compliance with Ethical Considerations (Criterion 4)

Monitoring Capacity (internal or external) (Criterion 4)

Quality control (Criterion 4)

Awareness of regulatory requirements for medicines development (Criterion 5)

Training received/given (Criterion 5)

Involvement of patients in either protocol design, or patient information package, or prioritisation of needs for clinical trials (Criterion 6)

Following the publication of research quality requirements, a set of recognition criteria, which have to be fulfilled to become a member of ENPR-EMA were agreed [8]. So far, 32 networks and centres (of 62 identified networks) submitted ***self-assessment reports ***indicating whether or not they fulfill the agreed minimum criteria.

Sixteen networks (26% of 62 identified networks) fulfilled all minimum criteria and became therefore members of ENPR-EMA. Three networks still having to clarify some issues before fulfilling criteria; the other networks currently not fulfilling minimum criteria.

The FIMP-MCRN [9-11], member of ENCEPP (European Network of Centres for Pharmacoepidemiology and Pharmacovigilance), became member of the ENPR-EMA responding satisfactorily to all the points of the self-assessment report [12].

The FIMP-MCRN was established in 2003 with the aim of developing competence, infrastructure, networking and education for paediatric clinical trials. It has been created to improve international standards of ethics and scientific quality by means of an agreement between individuals co-operating for the same objectives, goals and quality standards. The FIMP - MCRN have developed and improved expertise in: observational and epidemiological studies; efficacy studies; safety studies according to the Law - M.D. 139/2001 (this Law expresses the possibility of family pediatricians to perform Phase 3 and Phase 4 in their ambulatories) [11].

The FIMP-MCRN has got a system of management of the quality, a Quality Control, a Quality Assurance and a traceability, a transparency and a data safety. Furthermore, ad hoc training programs have incremented knowledge about clinical trials in Family Paediatricians to build up the necessary competencies, to facilitate co-operation, and to avoid duplication of studies [11].

## List of networks members of EnprEMA and other networks not yet fulfilling all criteria [12]

Networks fulfilling all minimum criteria:

**ITCC **(Innovative Therapies for Children with Cancer)

**Newcastle-CLLG **(Newcastle CCLG Pharmacology Studies Group)

**IBFMSG **(Network of National Study Groups for the treatment of hematological malignancies)

**ECFS - CTN **(European Cystic Fibrosis Society-Clinical Trials Network)

**MCRN-UK **[National Institute for Health Research (NIHR) Medicines for Children Research Network (MCRN) United Kingdom]

**FINPedNet **(Finnish Investigators Network for Pediatric Medicines)

**EUNETHYDIS **(the European Network for Hyperkinetic Disorders).

**EPOC **(A European Paediatric Oncology off patent medicines Consortium)

**FIMP-MCRN **(Family Paediatricians - Medicines for Children Research Network)

**UKPVG **(United Kingdom Paediatric Vaccines Group)

**PENTA **(Paediatric European Network for the Treatment of AIDS)

**MICYRN **(Mother Infant Child Youth Research Network)

**Scotmcn **(Scottish medicines for children network)

**GNN **(German Neonatal Network)

**PRINTO **(Pediatric Rheumatology International Trials Organisation)

**MCRN-NL**(Medicines for Children Research Network - The Nederland)

Networks still having to clarify some issues before fulfilling criteria:

**EBMT **(European Group for Blood and Marrow Transplantation)

**CLG **(Children Leukemia Group)

**CICPed **(Paediatric Network of Clinical Investigation Centers)

Networks currently not fulfilling minimum criteria:

**BPDN **(Belgian Pediatric Drug Network)

**AMIKI **(The Paediatric Trial Network)

**EuroNeoNet **(European Neonatal Network)

**JSWG of PRES **(Juvenile Scleroderma Working Group Pediatric Rheumatology European Society)

**Neo-circulation **(Network in Neonatology)

**PENTI **(Paediatric European Network for the Treatment of Infection)

**RIPPS **(Réseau d'Investigations Pédiatriques des Produits de Santé)

**IPCRN **(Irish Paediatric Clinical Research Network)

**BLF **(Swedish Peadiatric Society)

**NCCHD **(National Center for Child Health and Development)

**IPTA **(International Pediatric Transplant Association)

**FUTURENEST CLINICAL RESEARCH ESPGHAN **(European society of Gastroenterology, Hepatology and Nutrition)

## Third Workshop on European Network of Paediatric Research at the EMA (ENPR-EMA)

Once that was set up on ENPR-EMA with the inclusion of the 16 networks, it was considered necessary to think about the next step for its completion which provides for the organization of the Third Workshop on European Network of Paediatric Research at the EMA [13].

The Workshop will be held in London from 11 to 12 March 2011 at the headquarters of the EMA and will be organized into two separate days.

The first day of the workshop will be dedicated to discussions between networks to establish the Coordinating Group of ENPR-EMA and to discuss and to define priority tasks of the coordinating group.

The second day is being organised with the assistance of TOPRA (The Organisation for Professionals in Regulatory Affairs) where ENPR-EMA will be introduced to all stakeholders, particularly patient organisations and clinical researchers and pharmaceutical industry staff responsible for paediatric studies. The aim is to define the expectations of the various stakeholders and to offer the possibility for industry engaged in paediatric clinical trials and networks to interact.

The main objective is to conduct clinical trials of pediatric medicines conducted on an ethical basis, with the methodological quality necessary and with the character of independent research (the principal investigator processes the research project and is owner of the data obtained).

## Conclutions and future prospects

The EUPR wants to link together and coordinate existing networks, establish a platform for communication and exchange of information between European networks and share the skills and expertise in order to cut to shape and influence future development in paediatric research.

The purpose of this article is to disseminate knowledge of the structure and the goals of ENPR-EMA and to highlight that networking is necessary to build up the competence, to make co-operation easier and to avoid duplication of studies.

The Family Paediatricians Medicines for Children Research Network (FIMP-MCRN), established with the aim of developing competence, infrastructure, networking and education for paediatric clinical trials, became member of this European network. Quality and ethicality of research are becoming important end-points of Family Paediatricians during their education and during the sponsoring of health and prevention in the paediatric age groups. These possibilities could grant new paediatric medicine research opportunities and challenges for FIMP-MCRN and could guarantee further exchange of experience and competence, being an important wedge in the cultural mosaic of paediatric research [9].

## Competing interests

The author declares that he has no competing interests.

## Authors' contributions

EN conceived of and wrote the manuscript.

## Author Details

Ettore Napoleone is the Chair of FIMP- MCRN (Family Paediatricians -Medicines for Children Research Network), Member of Paediatric Working Group AIFA (Italian Medicines Agency) and Past President S.I.P (Italian Society of Paediatrics) - Molise
